# The Spatial Analysis on Hemorrhagic Fever with Renal Syndrome in Jiangsu Province, China Based on Geographic Information System

**DOI:** 10.1371/journal.pone.0083848

**Published:** 2014-09-10

**Authors:** Changjun Bao, Wanwan Liu, Yefei Zhu, Wendong Liu, Jianli Hu, Qi Liang, Yuejia Cheng, Ying Wu, Rongbin Yu, Minghao Zhou, Hongbing Shen, Feng Chen, Fenyang Tang, Zhihang Peng

**Affiliations:** 1 Department of Epidemiology & Biostatistics, School of Public Health, Nanjing Medical University, Nanjing, Jiangsu, China; 2 Jiangsu Province Center for Disease Control and Prevention, Nanjing, Jiangsu, China; University of Texas Medical Branch, United States of America

## Abstract

**Background:**

Hemorrhagic fever with renal syndrome (HFRS) is endemic in mainland China, accounting for 90% of total reported cases worldwide, and Jiangsu is one of the most severely affected provinces. In this study, the authors conducted GIS-based spatial analyses in order to determine the spatial distribution of the HFRS cases, identify key areas and explore risk factors for public health planning and resource allocation.

**Methods:**

Interpolation maps by inverse distance weighting were produced to detect the spatial distribution of HFRS cases in Jiangsu from 2001 to 2011. Spatio-temporal clustering was applied to identify clusters at the county level. Spatial correlation analysis was conducted to detect influencing factors of HFRS in Jiangsu.

**Results:**

HFRS cases in Jiangsu from 2001 to 2011 were mapped and the results suggested that cases in Jiangsu were not distributed randomly. Cases were mainly distributed in northeastern and southwestern Jiangsu, especially in Dafeng and Sihong counties. It was notable that prior to this study, Sihong county had rarely been reported as a high-risk area of HFRS. With the maximum spatial size of 50% of the total population and the maximum temporal size of 50% of the total population, spatio-temporal clustering showed that there was one most likely cluster (LLR = 624.52, P<0.0001, RR = 8.19) and one second-most likely cluster (LLR = 553.97, P<0.0001, RR = 8.25), and both of these clusters appeared from 2001 to 2004. Spatial correlation analysis showed that the incidence of HFRS in Jiangsu was influenced by distances to highways, railways, rivers and lakes.

**Conclusion:**

The application of GIS together with spatial interpolation, spatio-temporal clustering and spatial correlation analysis can effectively identify high-risk areas and factors influencing HFRS incidence to lay a foundation for researching its pathogenesis.

## Introduction

Hemorrhagic fever with renal syndrome (HFRS) is caused by hantaviruses comprising 23 identified species, while more than 30 as-yet-undetermined species are rodent-borne pathogens with a global distribution [1]–[2]. Human beings are believed to be infected by hantaviruses through inhalation of contaminated aerosols shed in the excreta, saliva, and urine of infected rodents [3]. Old-world rodents carry viruses that cause HFRS, while new-world rodents carry viruses that cause hantavirus pulmonary syndrome (HPS) [4]. HFRS is highly epidemic in China, accounting for 90% of total reported cases worldwide [5]. Although integrated control measures such as rodent control, environmental management, and vaccination have currently been implemented, HFRS remains a severe public health problem in mainland China; more than 10,000 human cases are diagnosed annually [6]. The incidence of HFRS shows high variability at both rural and urban levels [7]. Jiangsu, a highly developed coastal province, is one of the most severely affected provinces in China [8]. HFRS has spread to almost every county in Jiangsu since the 1980s, when the first HFRS case in Jiangsu was found [9]. Previous epidemiological surveys revealed the distribution of HFRS cases differed considerably from place to place [9].

In recent years, experts at home and abroad have focused on the aetiology, epidemiology and pathogenic mechanisms of HFRS, and have made substantial progress. Clement Jan et al. found that the infection rate of hantaviruses was closely associated with global warming [10]. A recent research report, which was the first to document an association between HFRS and PM_10_ levels, indicated that PM_10_ might be an important factor in HFRS infection [11]. The current study conducted an ArcGIS10.0-based spatial analysis involving the inverse distance weighting interpolation (IDW), spatio-temporal clustering and spatial correlation analysis to better comprehend space-time distribution patterns of HFRS cases in Jiangsu from 2001 to 2011, to find influencing factors of HFRS for public health interventions and to lay the groundwork for future research on these influencing factors.

## Materials and Methods

### Study area and data collection

The study area consisted of all 106 counties in Jiangsu province. Jiangsu is located at 116.60°∼121.67° east longitude and 31.01°∼34.89° north latitude on the central coast of China and has an area of 102.6 thousand square kilometers. Study samples were composed of all HFRS cases from 106 counties in Jiangsu from 2001 to 2011. Data that was not accessible to the general public were obtained from the Jiangsu provincial Center for Disease Control and Prevention (http://www.jshealth.com/). The work was approved by the ethical committee of Nanjing Medical University (“F”, “CH”, “Nanjing Med U”, “FWA00001501”, “NANJING”, 11/21/2004), and IRB (Institutional Review Board) approval was obtained prior to initiating the study. The authors have read and abided by the statement of ethical standards for manuscripts submitted to PLoS One.

### Spatial autocorrelation

Spatial autocorrelation was applied to describe the similarity of geographically proximate units. In this study, general spatial autocorrelation was used to explore the characteristics of the spatial distribution of HFRS cases, the results of which determined the presence of spatial clustering independent of location.

### Creation of interpolation maps

The IDW was applied to estimate the value of every point according to the principle that proximate sample points have similar values. In other words, the authors used measured values of the sample points to predict estimated values of every point in the study, then evaluated the error and variability of those predictions [12]. The researchers obtained the spatial distribution of HFRS cases in the overall region of Jiangsu from the limited spatial sample points by IDW. In this study, IDW was used to make interpolated maps using the data of HFRS from 2001 to 2011 in Jiangsu.

Our aim was to detect whether or not the epidemic was aggregated at the provincial level. General Moran's *I* was most often used, with the coefficient calculated as follows:
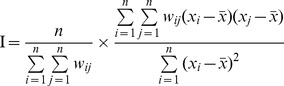



where *n* is the numbers of counties, *x_i_* and *x_j_* the observations from unit *i* to unit *j* with regard to the phenomenon *x* (the indicator of autocorrelations). *w_ij_*(d) represents the adjacent weight matrix from the distance d. If the unit *i* for regional data is adjacent to unit *j*, then *w_ij_*(d) is 1, otherwise it is 0. The *I* index was compared with the critical value of *Z*. If p<0.05, then Moran's *I*≠0, which means that the area indeed had an aggregation of HFRS infections.

### Spatio-temporal clustering

A spatio-temporal clustering analysis is defined by a cylindrical window with a circular (or elliptical) geographic base and a height corresponding to time [13]. To formulate the dynamic size and position of the cylindrical window, the center of the window moves according to the geographic union center and the radius changes continuously from zero up to the maximum radius, never including more than 50% of the total population. Every moment the radius changes, the log likelihood ratio (LLR) is calculated according to the difference between HFRS cases inside and outside the window. The formula for calculating the LLR is as follows: 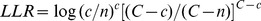: where C is the total number of HFRS cases, c is the observed number of HFRS cases inside the window and n is the expected number of HFRS cases inside the window.

The window with maximum LLR had the highest intensity of anomalies and was determined to be most likely to be a spatio-temporal cluster area. That is to say, the cluster was least likely to be clustered due to chance. For each window, a Monte Carlo simulation was used to calculate p to test the null hypothesis that the relative risk (RR) of HFRS was the same between any counties or any group of counties and remaining counties [14]. If the p-value of LLR was less than 0.05, the differences in relative risk (RR) between inside and outside the scan window and the abnormality degree of the incidence were considered statistically significant. In this study, a retrospective spatial cluster analysis for higher incidence was used. The maximum window radius was set to be smaller than 50% of the total population to find possible clusters.

### Spatial correlation analysis

The spatial correlation analyses in this study included univariate analysis and a spatial regression model that combined all underlying factors. Spatial regression analysis was used to detect potential relationships between the spatial distribution of a disease and independent variables (environmental factors such as water, air, soil, socioeconomic factors, etc.) determined by geography [15]. In order to avoid including influencing factors with no statistical significance, potential influencing factors were analyzed first by univariate analysis. Then, to avoid collinearity among the influencing factors with statistical significance, a multi-collinearity test was conducted on them. There have been a few reports suggesting that the incidence of HFRS is likely to be influenced by a complex combination of factors, including environment factors and climate factors, rather than by a single foci pathogenic factor [16]–[18]. Eleven risk factors for the spatial correlation analysis were selected from the previous reports including the annual mean temperature; the amount of water vapor; elevation; the normalized difference vegetation indexes (NDVI) for spring, summer, autumn and winter; and the distribution of lakes, rivers, railways and highways.

## Results

### General spatial autocorrelation

The research group found that the general Moran indexes of HFRS cases in all counties in Jiangsu were all greater than zero (Table 1). In the normal distribution hypothesis, the result of the Moran's I test showed spatial autocorrelation was highly significant, which indicated that HFRS cases in Jiangsu from 2001 to 2011 were significantly spatially autocorrelated. This revealed that the similarity of the observations in any two counties showed a negative correlation with the distance between the corresponding counties. This corresponds to the precondition of IDW.

**Table 1:** The results of the analysis on HFRS cases by general spatial autocorrelation.

Year	Z-score	P-value	Moran's Index	Cluster
2001	4.065	0	0.391	Highly clustered
2002	4.069	0	0.426	Highly clustered
2003	4.063	0	0.396	Highly clustered
2004	3.829	0	0.389	Highly clustered
2005	7.679	0	0.806	Highly clustered
2006	4.099	0	0.419	Highly clustered
2007	3.749	0	0.342	Highly clustered
2008	3.299	0	0.318	Highly clustered
2009	3.762	0	0.361	Highly clustered
2010	3.142	0.002	0.302	Highly clustered
2011	3.568	0	0.363	Highly clustered

### The inverse distance weighted interpolation

**Figure 1:**
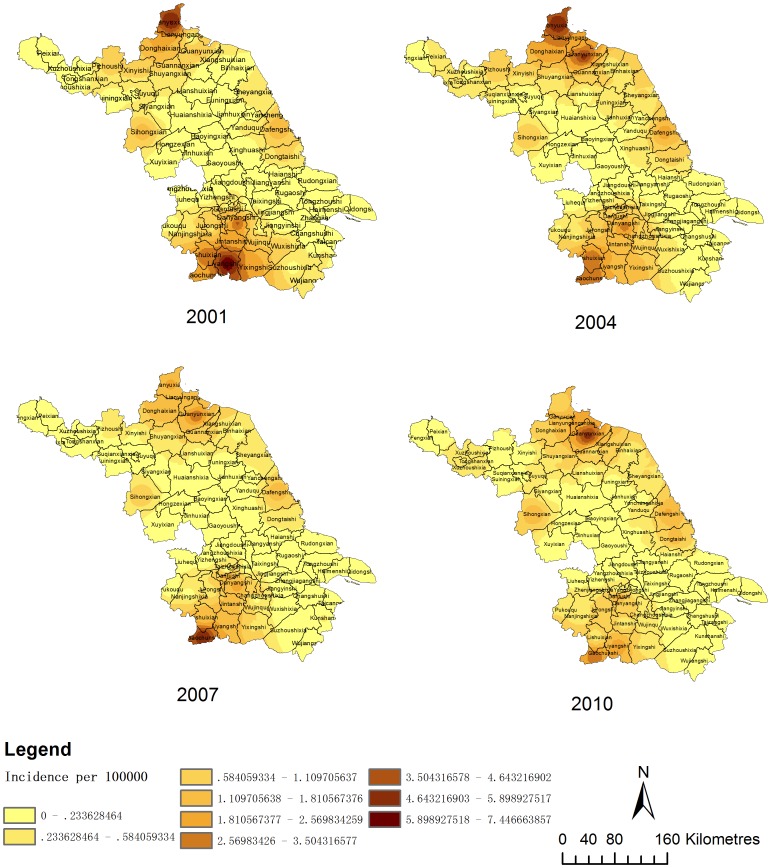
Interpolated maps of HFRS by IDW in Jiangsu in 2001, 2004, 2007 and 2010. The incidence of HFRS per 100,000 residents is shown in the map. The incidence of HFRS has a positive relationship with color depth.

Interpolated maps of HFRS incidence in Jiangsu from 2001∼2011 were produced by IDW. Darker colors were associated with higher incidence. The maps revealed that high-risk areas were distributed in the following counties: Ganyu, Lianyungang, Ganyu of northeastern Jiangsu, Gaochun and Liyang of southwestern Jiangsu, Dafeng of central-eastern Jiangsu and Sihong of central-western Jiangsu (Figure 1).

At the same time it was discovered that in addition to southwestern, northeastern and central-eastern Jiangsu, HFRS had also spread to central-western Jiangsu. However, the area of central-western Jiangsu where HFRS infections occurred was relatively small compared with the areas in southwestern, northeastern and central-eastern Jiangsu. This showed that the range of the region where HFRS cases were distributed had expanded, but in general southwestern and northeastern Jiangsu were the main areas affected and the general incidence of HFRS had dropped dramatically.

### Spatio-temporal clustering

Spatio-temporal clustering of HFRS cases in 2001∼2011 in Jiangsu showed that HFRS was not distributed randomly in space-time. One most likely cluster and one second-most likely cluster were identified with a maximum spatial size of 50% of the total population and a maximum temporal size of 50% of the total population. The most likely cluster (LLR = 624.52, P<0.0001, RR = 8.19) was in an 86.38 km radius around Gaochun located at latitude 31.36 north, longitude 118.99 east from 2001 to 2004. It encompassed seven counties (Gaochun, Lishui, Liyang, Jintan, Jurong, Yixing, Danyang). A second-most likely cluster (LLR = 553.97, P<0.0001, RR = 8.25) was in a 65.76 km radius around Ganyu located at latitude 34.90 north, longitude 119.006311 east from 2001 to 2004. This cluster encompassed four counties: Ganyu, Lianyungang, Donghai and Ganyun (Table 2).

**Table 2:** Spatio-temporal clustering on HFRS in Jiangsu from 2001 to 2011.

Spatio-temporal clusters	The most likely cluster	The second-most likely cluster
Center	Gaochun	Ganyu
Geographic coordinates of the center of clusters	(31.36N, 118.99E)	(34.90N, 119.01E)
Radius(Km)	86.38	65.76
Time	2001∼2004	2001∼2004
Number of counties included	7	4
LLR^a^ value	624.52	553.97
P-value	<0.0001	<0.0001
RR^b^ Value	8.19	8.25

a. LLR: Log likelihood ratio. b. RR: Relative risk.

The most likely cluster and the second-most likely cluster were distributed in northeastern and southwestern Jiangsu from 2001 to 2004, which showed that HFRS cases were mainly distributed in northeastern and southwestern Jiangsu and also that the prevalence of HFRS had dropped since 2004. This is in line with the results of IDW. It seems practical, therefore, to apply IDW and spatio-temporal clustering to an analysis of spatio-temporal dynamic trends of HFRS in Jiangsu.

### Spatial correlation analysis

The results of the univariate analysis on the potential relationships between the incidence of HFRS and the annual mean temperature; average amount of water vapor; average elevation; distances to railways, highways, rivers and lakes; and NDVI values are shown in Table 3. Results indicated that the incidence of HFRS had a weak correlation with annual mean temperature, average amount of water vapor, elevation and seasonal NDVI; and a strong correlation with distances from railways, highways, rivers and lakes (Table 3). The results of multicollinearity tests of the four factors which were in strong correlation with the incidence of HFRS showed that the four factors had no linear relation (Table 4).

**Table 3:** The results of single factor analysis between the incidence of HFRS and potential influencing factors in Jiangsu from 2001 to 2011.

	Curve fitting equation	R^2^	F	P
Temperature	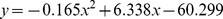	0.036	1.219	0.302
The amount of water vapor	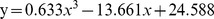	0.126	4.623	0.013
Elevation	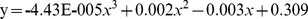	0.039	0.852	0.471
Distance to railway	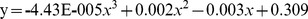	0.998	2524.638	0.000
Distance to highway	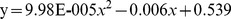	1.000	9304.654	0.000
Distance to river	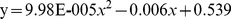	0.999	1766.491	0.000
Distance to lake	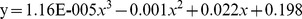	0.999	3651.710	0.000
NDVI^a^ for spring	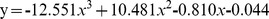	0.030	0.659	0.580
NDVI for summer	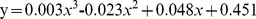	0.016	0.334	0.794
NDVI for autumn	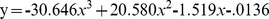	0.021	0.455	0.715
NDVI for winter	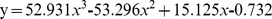	0.032	0.689	0.562

a. NDVI: normalized difference vegetation index.

**Table 4:** Collinearity diagnostics.

Model Dimension	Eigenvalue	Condition Index	Variance Proportions				
			(Constant)	Railway	Highway	River	Lake
1	2.603	1.000	0.05	0.04	0.05	0.03	0.05
2	1.037	1.584	0.01	0.14	0.02	0.40	0.09
3	0.644	2.010	0.05	0.01	0.20	0.29	0.45
4	0.372	2.645	0.02	0.48	0.72	0.28	0.08
5	0.344	2.752	0.87	0.32	0.01	0.00	0.33

Assuming the incidence of HFRS as a dependent variable and the distance from railways, highways, rivers and lakes as independent variables, the regression equation could be established. The regression equation was as follows:
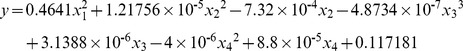




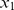
,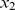, 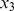 and 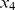 indicate the distances from railways, highways, rivers and lakes, respectively. The significance test and the goodness-of-fit test were applied to the regression equation. The results were 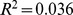,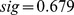 and 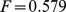, indicating that the equation had a high level of statistical significance and a low level of goodness of fit.

## Discussion

Geographic information systems (GIS) are widely used in epidemiology research for their advantages in accurately showing the spatial distribution of diseases, revealing spatial clusters of diseases and detecting potential risk factors contributing to disease transmission [19]–[20]. However, the application of IDW must meet the condition that the sample sites are efficiently distributed densely enough to cover all areas for which predictions are sought [21]. In this study, the sample of 106 sites appears to be large enough to cover all areas, and the sites were distributed evenly. Therefore, it was suitable to apply IDW. In figure 1, IDW interpolation maps show that HFRS cases are mainly distributed in Ganyu, Lianyungang, and Guanyun counties of northeastern Jiangsu, Gaochun and Liyang counties of southwestern Jiangsu, and Dafeng in central-eastern Jiangsu and Sihong in central-western Jiangsu. This finding is almost entirely in line with the results of Rongqiang Zu's study [9], with the exception of Sihong and Dafeng. Among the high-risk areas, Sihong was the only one that had rarely been reported in previous studies. Over the past several years, more land in Sihong has become farmland [22]–[23], which provides an abundance of food and suitable habitats for rodents and has resulted in an increasing number of rodents. The growth in the rodent population increases the chances of human contact with rodents and their excreta. Therefore, the increased area of farmland may be an important factor that contributed to the prevalence of HFRS in Sihong.

**Figure 2:**
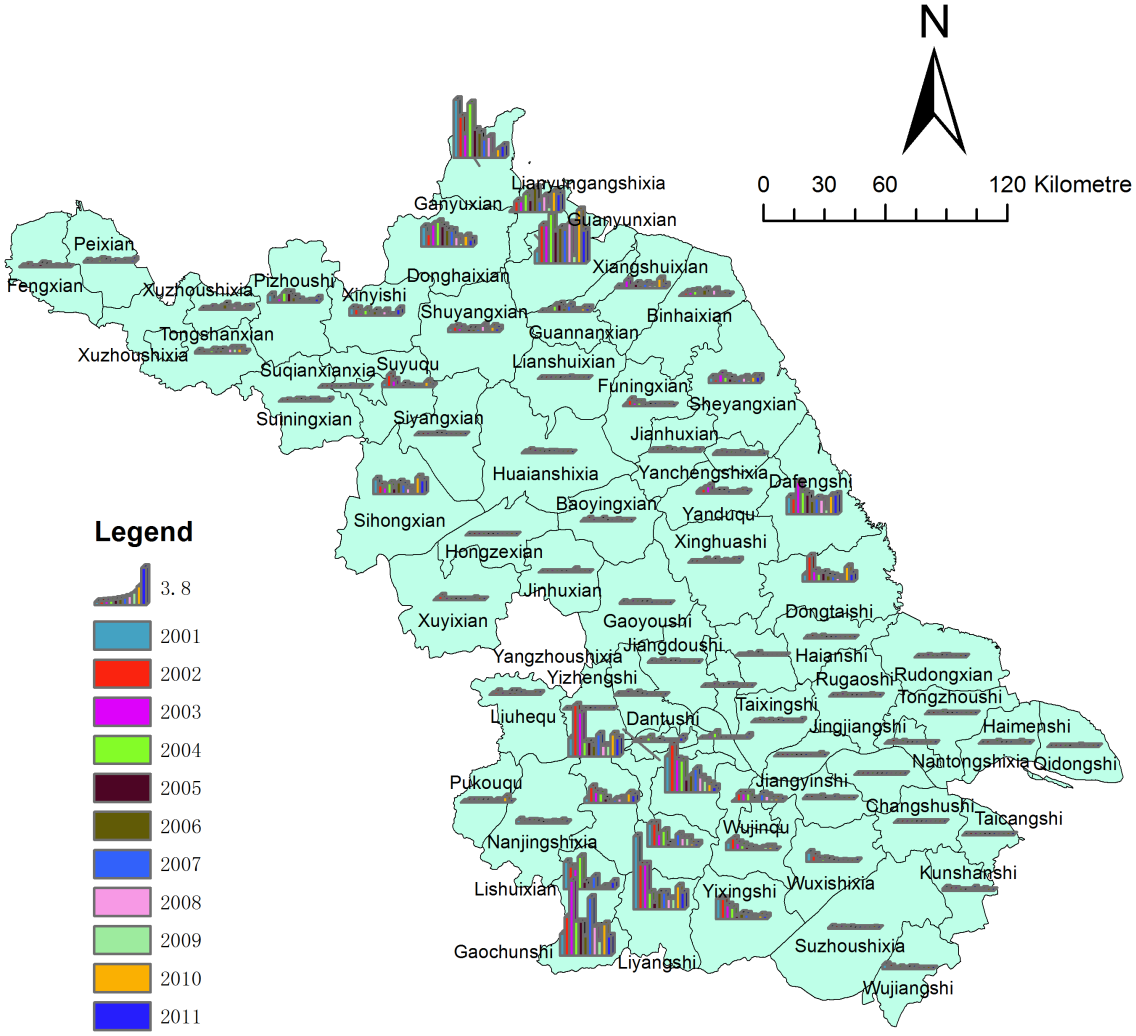
The density of HFRS cases based on counties in Jiangsu from 2001 to 2011. The mean annual incidence per 100,000 residents in each county of Jiangsu is shown. Different colors represent different years.

**Figure 3:**
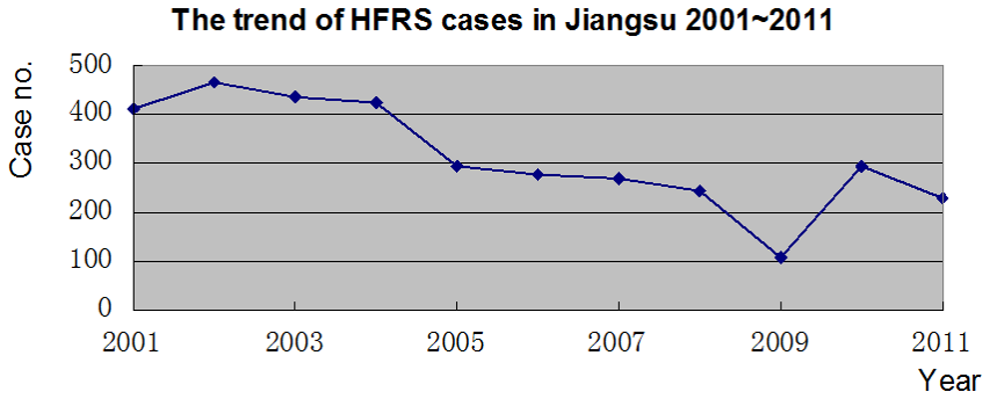
The trend of the number of HFRS cases in Jiangsu from 2001 to 2011. Each point represents the number of HFRS cases in a specific year. All of the points are lined to indicate the trend of the HFRS cases in Jiangsu.

The application of spatio-temporal clustering to computing the difference between incidence inside and outside the circular window lowers the possibility of pre-selection bias, which occurs when researchers determine the range of the study area and the center of the circle [24]. In this study, spatio-temporal clustering was used to determine the distribution pattern of HFRS cases and to identify key areas for future planning and resource allocation. Jiangsu can be divided into four parts according to its terrain: the western hilly region, the northwestern low hilly region, the southwestern low hilly region and the plains region. The spatio-temporal clustering results showed two cluster regions distributed in the northwestern and southwestern low hilly regions, respectively. The findings of HFRS clustering are consistent with an earlier study by Hualiang Lin, et al. [25]. The low hilly regions have more forest than the plains regions and more crops than the hilly regions. There is more forest in the northwestern and southwestern hilly regions, which provides a habitat for rodents, and the abundant crops provide them with sufficient food. Therefore, the chances of human contact with rodents and their excreta increase. At the same time, it was found that both cluster regions occurred in 2001∼2004 rather than in different periods of time. There were two clusters from 2001 to 2004 whereas there were no clusters from 2005 to 2011, which showed that the prevalence of HFRS decreased from 2005 to 2011 compared to the period from 2001 to 2004. This corresponds to the trend that the number of people infected with HFRS and patient density based on counties in Jiangsu declined from 2001 to 2011 (Figure 2 and Figure 3).

The spatial correlation analysis found that the incidence of HFRS was highly correlated with distances to highways, railways, rivers and lakes. Research has shown that the distance to a railway may cause a change in rodent density to a certain extent [26]. It is possible that railway transportation provides a suitable living environment and ample food for rodents.

In spite of the insights gained, the limitations of the study should also be acknowledged. Although spatio-temporal scan statistics can avoid a pre-selection bias successfully, a population shift bias may occur due to the lack of consideration of population growth. Therefore, both the number of HFRS cases and the unevenness of the population distribution among counties at different times can contribute to the occurrence of clusters [27]. If the population of one region increases faster than that of other regions, population shift bias may occur. The time span of this study is 11 years, during which the rate of population growth may have varied in different counties, so it is likely that population shift bias has occurred. In addition, this study only analyzed the causes of clusters of HFRS cases qualitatively instead of studying the quantitative relationship between possible influencing factors and the incidence of HFRS, due to a shortage of sufficient data about the environment, economy and geography. This may have reduced the study's rigor.
